# Scapular fracture: lower severity and mortality

**DOI:** 10.1590/S1516-31802008000300009

**Published:** 2008-05-01

**Authors:** Javad Salimi, Ali Khaji, Mojgan Karbakhsh, Soheil Saadat, Behzad Eftekhar

**Keywords:** Scapula, Fractures, bone, Multiple trauma, Injury severity score, Mortality, Escápula, Fraturas ósseas, Traumatismo múltiplo, Escala de gravidade do ferimento, Mortalidade

## Abstract

**CONTEXT AND OBJECTIVE:**

The presence of scapular fracture is believed to be associated with high rates of other injuries and accompanying morbidities. The aim was to study injury patterns and their overall outcomes in patients with scapula fractures.

**DESIGN AND SETTING:**

Cross-sectional study of trauma patients treated at six general hospitals in Tehran.

**METHODS:**

One-year trauma records were obtained from six general hospitals Among these, forty-one had sustained a scapular fracture and were included in this study.

**RESULTS:**

Scapular fracture occurred predominantly among 20 to 50-year-old patients (78%). Road traffic accidents (RTAs) were the main cause of injury (73.2%; 30/41). Pedestrians accounted for 46.7% (14/30) of the injuries due to RTAs. Falls were the next most common cause, accounting for seven cases (17.1%). Body fractures were the most common type of scapular fractures (80%). Eighteen patients (43.9%) had isolated scapular fractures. Limb fracture was the most common associated injury, detected in 18 cases (43.9%). Three patients (7.3%) had severe injuries (injury severity score, ISS ≥ 16) which resulted in one death (2.4%). The majority of the patients were treated conservatively (87.8%).

**CONCLUSIONS:**

Patients with scapula fractures have more severe underlying chest injuries and clavicle fractures. However, this did not correlate with higher rates of injury severity score, intensive care unit admission or mortality.

## RESUMO

**CONTEXTO E OBJETIVO:**

Acredita-se que a presença de fraturas escapulares esteja associada a alta freqüência de outras lesões e morbidades relacionadas. O objetivo foi avaliar o padrão de lesões e os desfechos gerais em pacientes com fraturas escapulares.

**DESENHO E LOCAL:**

Trabalho transversal com pacientes apresentando trauma, tratados em seis hospitais gerais em Teerã.

**MÉTODOS:**

Registros de trauma foram obtidos em seis hospitais gerais no período de um ano. Entre estes pacientes, 41 sofreram fratura escapular e foram incluídos no estudo.

**RESULTADOS:**

A faixa etária predominante para fratura escapular foi 20-50 anos, sendo 78%. Acidentes automobilísticos (AAs) foram a maior causa de lesão, em 73,2% (30/41). Observou-se também que os acidentes com pedestres correspondiam a 46,7% (14/30) das lesões devidas a AAs. Quedas foram a segunda causa mais freqüente, com sete casos (17,1%). Fraturas do corpo estavam o tipo mais freqüente das fraturas escapulares (80%). Dezoito pacientes (43,9%) apresentaram apenas fratura escapular. Fraturas de extremidades foram as lesões mais comumente associadas, observadas em 18 (43,9%). Três pacientes (7,3%) tiveram lesões graves (escala de gravidade das lesões ≥ 16), o que resultou em um caso (2,4%) de óbito. A maioria dos pacientes foi tratada de forma conservadora (87,8%).

**CONCLUSÕES:**

Pacientes com fraturas escapulares tiveram lesões torácicas e fraturas de clavícula basicamente mais graves. Porém, não houve correlação com maiores índices da escala de gravidade das lesões, admissão na unidade de terapia intensiva ou mortalidade.

## INTRODUCTION

Fracture of the scapula is an infrequent injury, with an incidence of 0.4% to 1% of all fractures.^1,2^ The low incidence is attributed to its anatomical position and muscle envelope.^2-4^ The most common area of the scapula to be fractured is the body.^1,5^ Motor vehicle accidents are the main cause of scapular fractures, accounting for more than 50% of the cases.^3,5,6^ Significant force is required to fracture the scapula; thus, the presence of scapular fractures is believed to be associated with high rates of other injuries and accompanying morbidities. Associated injury has been reported in 35% to 98% of patients with scapular fractures.^1-3,5^ On the other hand, there are a few case reports of SF related to indirect trauma with less violent causes.^7-9^ Stephens et al.^10^ reported that scapular fractures are not a significant marker for greater general or eurovascular morbidity among blunt-trauma patients. Veysi^5^ reported that patients with scapular fractures have more severe underlying chest injuries and worse overall injury severity score (ISS) but that this did not correlate with a higher rate of intensive therapy before admission, duration of hospitalization, or mortality.

## OBJECTIVE

We studied injury patterns and overall outcomes among patients with scapular fracture.

## METHODS

This cross-sectional study was conducted between August 1999 and September 2000 in six of the largest university hospitals in Tehran, with high numbers of trauma patients. These hospitals are located in different parts of the city. In the absence of dedicated trauma centers, these university hospitals admit a significant number of severely injured patients.

Structured closed-question data checklists were used for the data gathering process. The checklists were completed by trained physicians visiting trauma patients in emergency rooms and wards around the clock. Data obtained included: patients’ demographics, prehospital care, medical and operative procedures performed in emergency rooms and wards (according to the International Classification of Diseases, ICD-9 coding), Glasgow coma scale (GCS), vital signs at the time of presentation to emergency rooms (ERs), injury severity score (ISS), duration of hospitalization, time spent in intensive care unit (ICU), source of reimbursement and outcome. The injury and mechanism of the accident were categorized based on ICD-10 (International Classification of Diseases, tenth revision).

Statistical analyses were performed using the Statistical Package for the Social Sciences (SPSS) software (version 10.0 for Windows). The chi-squared and Student’s t test were used for the statistical analysis. p-values less than 0.05 were regarded as statistically significant.

## RESULTS

During the study period, 8,420 trauma patients were admitted to the six general hospitals. Among these patients, 41 (0.48%) had sustained a scapula fracture (group 1) and the remainder (99.5%) formed the control group for the study (group 2). There was no difference between the two groups in terms of age and sex distribution (Table 1).

**Table 1:** Patients’ demographics. There was no significant difference significant between groups

	Group 1 (Scapular fracture)	Group 2 (No scapular fracture)
**Age**		
Mean	35.95	32.63
**Sex**		
(Male to female ratio)	34/7	6345/2034
**Glasgow coma scale**		
Mean	14.9	14.5
**Injury severity score**		
Mean	7	6.9

Overall, 33 patients (80%) sustained body scapular fractures (Type III), two patients (4.9%) sustained fractures of the glenoid and neck of the scapula (Type II) and four patients (9.8%) had fractures of coracoid process and acromion (Type I). Road traffic accidents (RTAs) were the most common cause of injury in both sets of patients, accounting for 70.7% of the patients in group 1 and 46.5% in group 2 (Table 2). Falls were the next most common cause (19.5% in group 1 versus 36.7% in group 2). Scapular fracture alone was detected in 36.6% (15/41) of the patients. The mean number of accompanying injuries was 1.5/person. Limb fracture was the most common associated injury, observed in 18 cases (43.9%), followed by head injury and thoracic injury with five cases each (12.2% each). The frequency of the accompanying injuries is listed in Table 3. The incidence of head injuries in the two groups was not significantly different (12.2% in group 1 versus 10.3% in group 2; p = 0.68) (Table 4).

**Table 2:** Mechanism of injury in patients with scapular fracture

Mechanism	Male (%)	Female (%)	Total (%)
**Road traffic accident**	26 (76.5)	4 (57.1)	30 (73.2)
Pedestrian	12	2	14
Car passenger	5	2	7
Motorcyclist	8	0	8
Bicycle rider	1	0	1
**Fall**	6 (17.6)	1 (14.3)	7 (17.1)
height ≥ 4 meters	2	0	2
height < 4 meters	2	0	2
stairs	1	1	2
falling to the ground	1	0	1
**Blunt object**	1 (2.9)	2 (28.6)	3 (7.3)
**Cutting**	1 (2.9)	- (0)	1 (2.4)
**Total**	**34**	**7**	**41 (100)**

**Table 3:** Frequency of associated injury in patients with scapular fracture

Type of injury	Number (%)
Skull fracture	6 (9.8)
Facial bone fracture	5 (8.2)
Brain injury	4 (6.6)
Clavicle fracture	7 (11.5)
Long bone fracture	22 (36.1)
Dislocation	8 (13.1)
Rib fracture	5 (8.1)
Hemothorax	2 (3.3)
Pelvic fracture	2 (3.3)
**Total**	**61 (100)**

**Table 4:** Comparison of injuries between the two groups

	Group 1	Group 2	Relative risk	p-value
Head injury	5 (12.2%)	859 (10.3%)		–
Spinal fracture	0	229 (2.7%)		–
Chest injury	5 (12.2%)	279 (3.3%)	4.03 (1.6-10.4)	0.002
Abdominal injury	0	177 (2.1%)		–
Upper limb fracture	14 (34.1%)	1982 (23.7%)		–
Lower limb fracture	7 (17.1%)	3282 (39.2%)		–
Humerus fracture	5 (12.2%)	455 (5.4%)		–
Pelvic fracture	2 (4.9%)	311 (3.7%)		–
Femur fracture	3 (7.3%)	1402 (16.7%)		–
Clavicle fracture	7 (17.1%)	132 (1.6%)	12.8 (5.6-29.5)	0.0001

The patients in group 1 sustained chest injuries more significantly than did group 2 (12.2% versus 3.3%; p = 0.002). Rib fractures were detected in all the patients with chest injuries and two cases had hemothorax. Overall, the incidence of upper limb fractures was not significantly different between the two groups (34.1% in group 1 versus 23.7% in group 2) but fractures of the lower limbs were significantly more frequent in group 2 than in group 1 (39.2% versus 17.1%, respectively). On the other hand, there was no significant difference in the incidence of humerus fractures between group 1 and group 2 (Table 4). Clavicle fractures were significantly more frequent in group 1 than in group 2 (17.1% versus 1.6%; p = 0.0001, respectively). There was no significant difference in the severity of injury between the patients in group 1 and group 2 (7 ± 4.5 versus 6.9 ± 9.5, respectively). Among the patients with scapular fractures, three patients (7.3%) had severe injuries (ISS ≥ 16). Body fractures occurred significantly more often in the patients with multiple injuries (p = 0.04). 2.4% of the patients with scapular fractures and 3.4% of those without scapular fractures required admission to the intensive care unit (p = 0.7). One patient (2.4%) from group 1 died due to the severity of injury.

Head injury was the main cause of death. The difference in mortality rate between group 1 and group 2 was not significant: 2.4% (n = 1) versus 3.2% (n = 267); p = 0.7, respectively.

Five patients (12.2%) with scapular fractures were treated operatively. The mean length of hospitalization was 6.9 days (range 1-36 days).

## DISCUSSION

Scapular fractures and their reports are rare. The scapula is protected by its position in the posterior of the thorax and is shielded by a thick layer of muscle. The 4.8 to 1 male-to-female ratio seen in this study parallels previous reports and reflects male predominance in traumatic events.^4,5^ Scapular fractures were detected in 0.48% of our patients and comprised 0.8% of all limb fractures, which was consistent with previous reports.^1,2^ In reports by Veysi et al.^5^ and Weening et al.^11^, patients with scapular fractures accounted for 6.8% and 3.7%, respectively. These differences may have resulted from patient selection, since they selected patients with ISS ≥ 16 and ISS ≥ 12, respectively. The trauma patient selection may be the main cause of this difference.

Among our patients, the most frequent location of scapular fractures was the body (80%), which was similar to previous reports.^1,4,5,10^ As in the previous reports,^1,5,6,10^ motor vehicle collisions and falls were the overwhelming causes of injury among our patients, accounting for 73.2% and 17.1%, respectively. Our results revealed that pedestrians made up a considerable number of the RTA victims with scapular fractures (46.7%), along with other regularly appearing figures in traffic accidents (motorcyclist, bicyclists, etc). This is in contrast with previous reports^12-14^ that indicated that the majority of RTA victims with scapular fractures were injured in events involving two individuals (especially car occupants and motorcyclists). This might have contributed towards the setting of our study, since pedestrians accounted for 21.3% (1700/8420) of all of our patients. Scapular fractures alone are uncommon injuries, and associated injuries have been reported in 75% to 98% of the cases in different series.^1,2,3,6,10,11^

In our study, the incidence of associated injuries (63.4%) was lower than in some previous reports. The reported incidence of limb injury or fracture in patients with scapular fractures varies from 22.6% to 100%.^5,15^ Limb fracture was the prominent accompanying injury among our patients (51.2%). In our study, there was no significant difference in the rate of limb fracture between the patients with scapular fractures and the control group. The limb injuries among our patients were restricted to fractures and dislocations and we did not find any vascular or nerve injuries in limbs. Weening et al.^11^ reported that patients with scapular fractures had a greater proportion of tibial and fibular fractures, significantly. Veysi et al.^5^ showed that there were no significant fractures of any part of long bones in patients with scapular fracture and the control group. Brown et al.^9^ reported that fractures of the femur were significantly more frequent in patients with scapular fracture.

We found that a significantly higher number of patients sustained clavicle fractures in group 1 than in group 2. This finding is similar to previous studies.^5,13,14^ Five patients (12.2%) with scapular fractures had chest injuries, giving rise to an 11.4% rate of associated injuries among them. This rate is less than what was reported in previous studies.^5,9,11^ The incidence of chest injury in patients with scapular fracture was significantly higher than in the control group, although these types of injuries (rib fracture and hemothorax) are not usually life-threatening. There was no significant difference regarding head injuries between the two groups of our patients. Although head injuries did not make up a large proportion of the accompanying injuries in patients with scapular fractures, they were the main cause of death in these patients. In contrast to the majority of previous reports,^5,11,13^ we did not find any spinal injuries, vertebral fractures or abdominal injuries among patients with scapular fractures.

The severity of injury among the patients with scapular fractures was not different from severity of injuries among control group (7 ± 4.5 versus 6.9 ± 9.5, respectively). Three of our patients with scapular fractures (7.3%) presented severe injuries (ISS ≥ 16). Two previous reports have compared the ISS between patients with scapular fractures and the control groups. The mean ISS in the reports by Veysi et al.^5^ and Weening et al.^11^ was 22.8 and 28.3 respectively. Since they selected patients with ISS ≥ 16 and ISS ≥ 12, respectively, the trauma patient selection may also be the main cause of this difference.

The difference in the mortality rate between group 1 and group 2 was not significant: 2.4% (n = 1) versus 3.2% (n = 267), respectively. This mortality rate of 2.4% in group 1 is less than the rates of 14.3% reported by Thompson et al.^6^, 9.7% reported by Armstrong et al.^14^ and 11.4% by Veysi et al.^5^ The lower mortality noted in our study might be related to the fact that our patients with scapular fractures presented lower severity and lower prevalence of accompanying injuries. It has been well shown that scapular fracture does not lead directly to death. Instead, the severity of the associated injuries would determine the mortality rates among patients with scapular fractures. In our study, the associated injuries among the patients with scapular fractures were not severe enough to be life-threatening for these patients.

## CONCLUSIONS

Patients with scapula fractures have more severe underlying chest injuries and clavicle fractures. However, this did not correlate with higher rates of injury severity score, intensive care unit admission or mortality.
